# Effects of auricular acupressure on test anxiety in medical students: a randomized parallel-group trial

**DOI:** 10.1186/s12909-023-04825-w

**Published:** 2023-11-07

**Authors:** Zinab Mosavi, Habibolah Khazaie, Maryam Janatolmakan, Shahab Rezaeian, Alireza Khatony

**Affiliations:** 1 School of Medicine, Kermanshah University of Medical Sciences, Kermanshah, Iran; 2 Sleep Disorders Research Center, Kermanshah University of Medical Sciences, Kermanshah, Iran; 3 Social Development and Health Promotion Research Center, Health Institute, Kermanshah University of Medical Sciences, Kermanshah, Iran; 4 Infectious Diseases Research Center, Kermanshah University of Medical Sciences, Kermanshah, Iran

**Keywords:** Acupressure, Test anxiety, Medical students, Shen men, Auricular

## Abstract

**Background:**

Test anxiety is a prevalent issue among students, including those in the medical field. The present study aims to examine the impact of auricular acupressure on reducing test anxiety specifically among medical students.

**Methods:**

In this single-blind randomized parallel-group trial, a total of 114 medical students from Kermanshah, Iran, were allocated into two groups: intervention and control. Each group consisted of 57 students. The data collection instruments included a demographic information form and the Sarason Anxiety Inventory. In the intervention group, bilateral auricular acupressure was administered on the Shen Men point for a duration of 10 min. On the other hand, the control group received bilateral auricular acupressure on the Sham point, located in the earlobe, as a placebo, also for 10 min.

**Results:**

The mean test anxiety scores in the Shen Men acupressure group exhibited a significant reduction from 18.4 ± 5.3 before the intervention to 13.3 ± 4.8 after the intervention (P = 0.001). Conversely, in the Sham acupressure group, the mean test anxiety scores showed no significant change, with values of 16.36 ± 6.4 before the intervention and 16.4 ± 6.1 after the intervention (P = 0.963). Prior to the intervention, the majority of participants in both the intervention group (87.7%) and control group (86.0%) exhibited moderate to severe levels of test anxiety. Following acupressure, a significant improvement was observed in the intervention group, with 52.6% of participants experiencing a reduction to mild anxiety levels (P = 0.001); however, no notable change in anxiety levels was observed in the control group. Furthermore, a statistically significant difference in anxiety intensity after the intervention was found between the two groups (P = 0.001).

**Conclusion:**

Shen Men auricular acupressure demonstrates efficacy in reducing test anxiety among medical students. However, to validate its effectiveness, further research using objective measures is warranted.

## Introduction

One common source of anxiety among students is examinations [1]. Test anxiety is a psychological condition that leads to feelings of anxiety during exams, thereby disrupting students’ performance [2, 3]. The severity of this condition varies among students and can manifest through physical, cognitive, behavioral, and emotional symptoms [4, 5]. The prevalence of test anxiety overall differs across societies, with estimates ranging from 30 to 58% [6–11]. Various factors contribute to test anxiety, including difficulty in retaining course material, time constraints during exams, inadequate exam preparation, fear of failure, challenging coursework, sleep disturbances on the night before exams, and poor time management [12–15].

Severe cases of test anxiety may necessitate therapeutic interventions [16]. Benzodiazepines are commonly prescribed drugs for treating test anxiety; however, they entail various side effects, including addiction, memory impairment/amnesia, and reduced concentration [17, 18]. Consequently, non-pharmacological approaches like acupressure have garnered increasing attention for managing test anxiety [19]. Acupressure, an alternative and complementary medicine modality, involves the application of pressure on specific points of the body, utilizing fingertips instead of needles, as in acupuncture [20]. While the precise mechanism of acupressure remains unclear [20, 21], this non-invasive technique regulates neurotransmitter levels, reduces the concentration of 5-hydroxytryptamine and adrenocorticotropic hormone in nerve pathways, and ultimately leads to anxiety reduction [22, 23].

The Shen Men point, located in the triangular fossa of the ear, is an acupressure point known for its relaxing and analgesic effects [24]. Existing research on the impact of acupressure on test anxiety is limited and has employed various pressure points, techniques, and durations [19, 21, 24, 25]. Klausenitz et al. (2016) conducted a study demonstrating the effectiveness of acupressure on multiple points, including those in the ear, abdomen, lung, subcortex, and adrenal gland, in reducing test anxiety [25]. Another study by Lee et al. (2021) highlighted the efficacy of auricular acupressure specifically on the Shen Men and endocrine points for reducing test anxiety [19]. McFadden et al. (2012) compared three methods—active acupressure, placebo acupressure, and soothing sounds—and found that all three methods were equally effective in reducing test anxiety [26]. Additionally, Joseph’s study (2015) indicated that self-administered acupressure at acupoint P6 for two minutes resulted in a reduction in test anxiety among students [21].

Test anxiety is a widespread issue among students, known to have detrimental effects on their physical, psychological, and academic well-being [4, 5, 27, 28]. Despite its significance, there is a scarcity of studies exploring the impact of acupressure on test anxiety, with variations in the areas targeted and techniques employed. Therefore, the present study aimed to examine the effects of auricular acupressure specifically at the Shen Men point on test anxiety among medical students. The study addressed the following research questions:

1. What is the prevalence of test anxiety among medical students?
2. What is the effect of auricular acupressure at the Shen Men point on test anxiety?
3. What is the effect of auricular acupressure at the Sham point (placebo) on test anxiety in the control group?
4. Is there a significant difference in test anxiety levels between the intervention and control groups after the acupressure intervention?

## Methods

### Study design

The current study employed a randomized, single-blind, parallel-group trial design, adhering to the guidelines outlined in the Consolidated Standards of Reporting Trials (CONSORT) criteria [29].

### Sample and sampling method

The study population consisted of medical students from Kermanshah University of Medical Sciences (KUMS), Iran. A total of 114 medical students were included in the study. Sample size estimation was conducted using the formula for comparing means between two independent groups. The sample size calculation was based on data from a study by Cho et al. (2021), where the mean anxiety scores in the intervention and control groups were reported as 41.1 ± 8.9 and 46.1 ± 9.9, respectively. Considering a test power of 80% and a confidence level of 95%, a total of 57 students were estimated for each group [30]. The inclusion criteria for participants in this study were as follows: having a minimum test anxiety score on the Sarason Anxiety Inventory (score 1), absence of coagulation diseases, absence of immunocompromised diseases, no history of thyroid diseases, psychiatric disorders, or epilepsy, not taking anti-anxiety or psychoactive medications, no prior experience with acupressure or acupuncture, and not being pregnant (for female participants). The exclusion criteria included participant refusal to participate, presence of skin rashes, active inflammation or infection in the ear area, and presence of skin lesions in the ear area.

The samples were selected for the study using convenience sampling and were then allocated to either the intervention or control group through block randomization. To carry out the randomization, an online tool (www.sealedenvelope.com) was utilized, generating 29 random blocks of four individuals each, for a total sample size of 114. The random sequences for each block were written on separate cards and placed inside opaque envelopes to ensure the concealment of the randomization process. Each envelope was assigned a number, and participants were allocated to their respective study groups based on the sequences indicated on the cards. For instance, envelope number one contained a card with the sequence AABB. The envelopes were sorted and numbered, and participants were assigned to their groups according to the letters on the cards. This process was continued until all participants were allocated. The participants were blinded to their group assignment to maintain blinding during the study.

### Study setting

The intervention was carried out in the examination hall of the Kermanshah School of Medicine, which serves as the venue for all medical students’ exams. The examination hall has a capacity to accommodate 200 students, providing a suitable environment for conducting the intervention in this study.

### Instruments

The data collection for this study involved the use of two tools: a demographic information form and the Sarason Anxiety Inventory. The demographic information form included questions related to participants’ age, gender, marital status, residence, and their grade point average from previous semester(s). The Sarason Anxiety Inventory, originally developed by Sarason et al. in 1975 [31], was used to assess test anxiety levels. The inventory has demonstrated good internal consistency, as confirmed by Raju et al. (2010) with an alpha coefficient of 0.84 [32]. The Persian version of the inventory has also been psychometrically evaluated in Iran, and its internal consistency has been confirmed with a Cronbach’s alpha coefficient of 0.74 [33, 34]. The Sarason Anxiety Inventory consists of 37 yes-no questions, with each correct answer assigned a score of one and each incorrect answer given a score of zero. The total score on the inventory can range from 0 to 37. Based on the obtained scores, participants are categorized into one of three anxiety levels: low anxiety (0–12), moderate anxiety (13–20), or high anxiety (21–37). Additionally, the mean score of test anxiety was calculated in the current study.

### Interventions

**Fig. 1:**
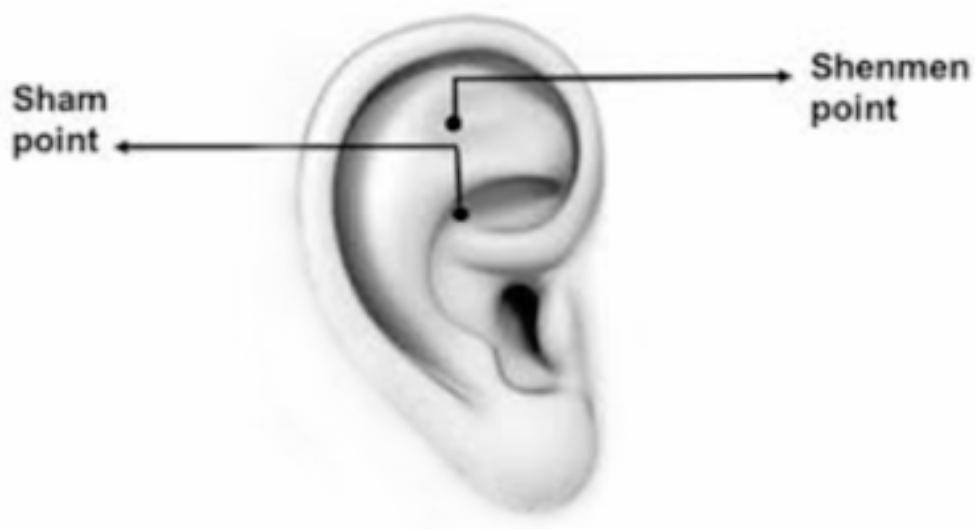
Location of Shen Men and Sham points

One hour before the test, the demographic information form, and the Sarason anxiety inventory were completed by the samples of both groups. The place of auricular acupressure in the experimental group was the Shen Men point. This point is located in the lateral triangular fossa of the auricles [24]. In the control group, a place called Sham was used, which is located in the earlobe. This point is a placebo and has no therapeutic effects [35] (Fig. 1).

The interventions in this study were conducted by two individuals, a male therapist who was a nursing graduate and a female therapist who was the first author of the study and a medical student. Prior to the study, both therapists received training in acupressure and their competence to perform the intervention was confirmed by an acupuncturist. The training involved seven one-hour face-to-face sessions conducted in the office of the acupuncturist.

After obtaining permission from the university, the researchers visited the test location, which was situated in the medical school, to carry out the sampling process. During this visit, the objectives of the study were explained to the students, and those who expressed willingness to participate were included in the study.

One hour before the test, both intervention and control groups completed the demographic information form and the Sarason Anxiety Inventory. The intervention group received auricular acupressure at the Shen Men point, which is located in the lateral triangular fossa of the auricles [24]. In contrast, the control group received acupressure at a point called Sham, located in the earlobe. The Sham point was used as a placebo and does not have any therapeutic effects [35]. (Fig. 1)

The selection of the Shen Men point for acupressure was based on several considerations. Firstly, the well-established calming effects of this point in traditional Chinese medicine have been widely recognized. Previous studies have also examined its effectiveness in alleviating pain and anxiety (19, 24). Secondly, the accessibility of the Shen Men point makes it feasible to utilize acupressure in the ear, provided its efficacy is confirmed (24). Furthermore, various acupressure points have been employed in studies focusing on the management of test anxiety (21, 25, 26). Hence, our current study aimed to assess the potential impact of acupressure at the Shen Men point on reducing test anxiety among medical students.

To perform the intervention, the therapists first washed their hands thoroughly to ensure hygiene. The subjects were then instructed to sit in a chair, adopting a relaxed position. In the intervention group, the therapists applied acupressure to the Shen Men acupoint bilaterally. The technique involved using the index finger to apply circular, gentle, and uniform pressure to the acupoint. The pressure was exerted for a duration of ten minutes.

**Fig. 2:**
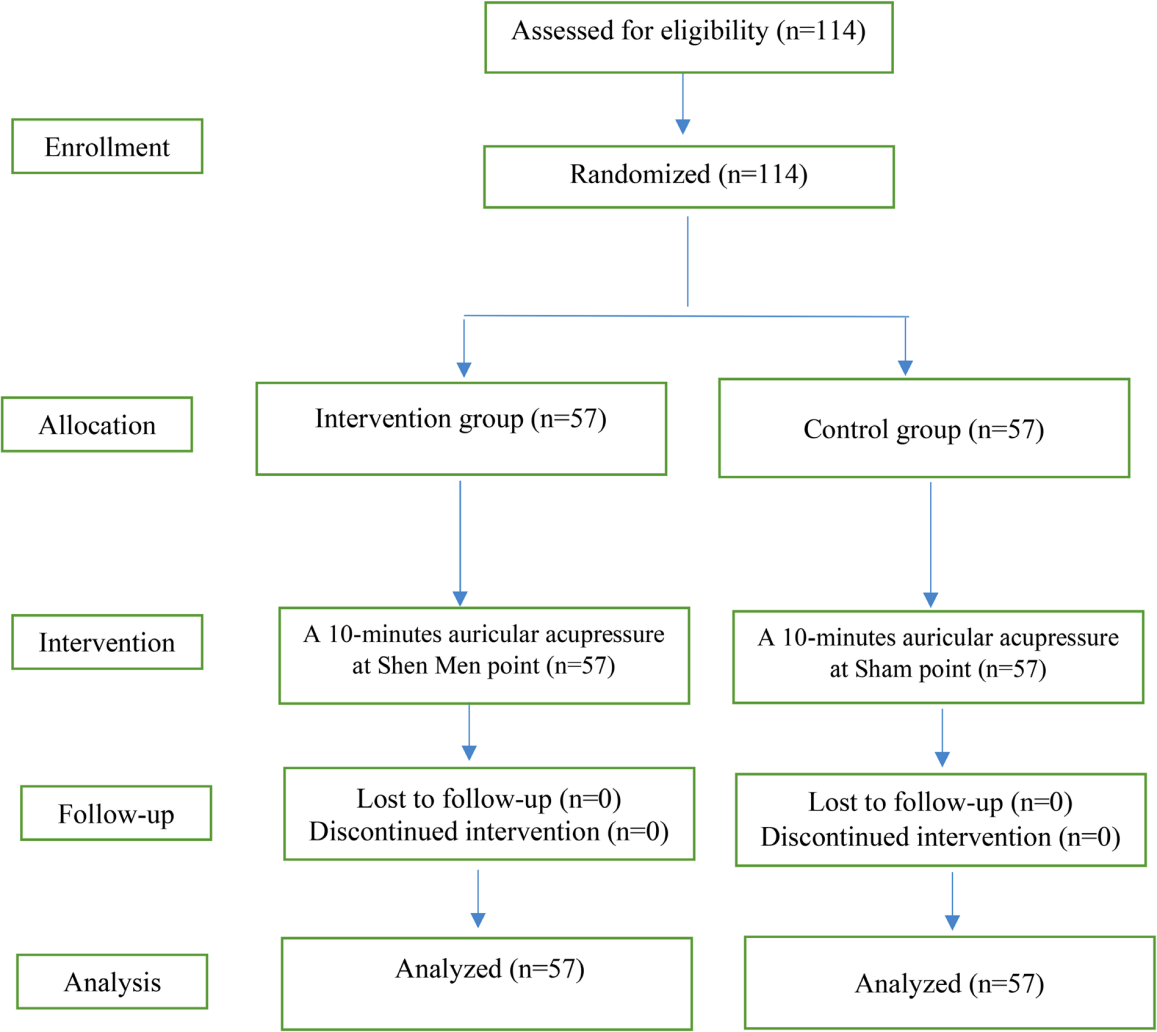
The CONSORT diagram of the study

In the control group, the Sham point was pressed in the same manner as the intervention group. Following the intervention, both the intervention and control groups completed the Sarason Anxiety Inventory to assess the effects of the intervention on test anxiety. It should be noted that no dropout occurred in the study (Fig. 2).

### Data analysis

The collected data were subjected to both descriptive and inferential statistical analyses. Descriptive statistics, including mean, standard deviation, frequency, and percentage, were employed to summarize the data. Inferential statistics, on the other hand, involved the use of several statistical tests, namely Chi-square, McNemar, independent t-tests, and paired t-tests. To compare the qualitative variables such as gender and marital status between the study groups, the chi-square test was utilized. For the quantitative variable of age, an independent t-test was employed for between-group comparisons. To assess the intensity of test anxiety before and after the intervention, the chi-square test was used to compare the scores between the two groups. Within each group, McNemar’s test was conducted to compare the anxiety scores before and after the intervention. Furthermore, the paired t-test was utilized to evaluate the mean test anxiety scores before and after the intervention within each group. Lastly, the independent t-test was applied to compare the mean test anxiety scores between the intervention and control groups. All statistical analyses were conducted using SPSS 18 software, with a confidence level set at 95%.

### Ethical considerations

The study with the code R.KUMS.MED.REC.1400.093 received approval from the Ethics Committee of Kermanshah University of Medical Sciences. Additionally, the study was registered and assigned the code IRCT20100913004736N24 in Iran’s clinical trials database at www.irct.ir on December 1, 2022. Prior to participation, the objectives of the study were thoroughly explained to all participants, and written informed consent was obtained from each of them. Confidentiality of their data and personal information was assured to all participants. All methods employed in the study adhered to relevant guidelines and regulations.

## Results

The study included a total of 114 medical students, resulting in a 100% response rate. The mean age of the participants was 21.9 ± 3.1 years. Among the participants, approximately half were male (50.1%, n = 58), and the majority were single (95.6%, n = 109) (Table 1).

**Fig. 3:**
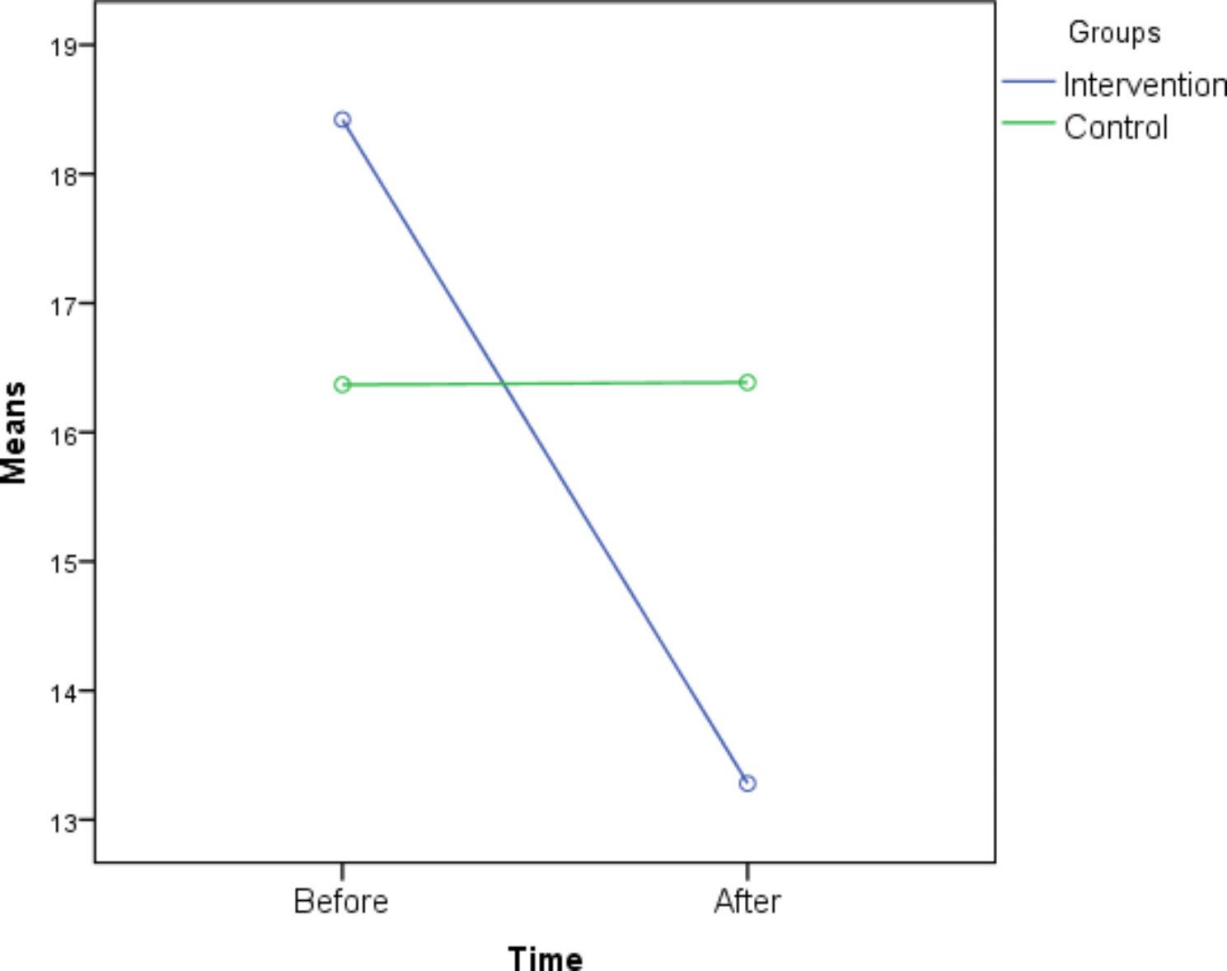
The comparison of mean test anxiety levels before and after the intervention in the study groups

In the experimental group, the mean anxiety scores before and after the intervention were 18.4 ± 5.3 and 13.3 ± 4.8, respectively. This difference was found to be statistically significant (P = 0.001), indicating a positive impact of the intervention (Table 2; Fig. 3).

Regarding the severity of test anxiety, the results revealed that 56.1% (n = 32) of the participants experienced moderate anxiety, while 31.6% (n = 18) had severe anxiety prior to the intervention. After the intervention, the number of participants experiencing mild anxiety increased to 52.6% (n = 30), and the number of individuals with severe anxiety decreased to 10.5% (n = 6). The McNemar’s test demonstrated a statistically significant difference in the intensity of test anxiety before and after the intervention (P = 0.001) (Table 3).

In the control group, the mean anxiety scores before and after the intervention were 6.4 ± 16.4 and 6.1 ± 16.4, respectively, indicating no significant change (Table 2; Fig. 3). Prior to the intervention, 59.7% (n = 34) of the participants had moderate test anxiety, while 26.3% (n = 15) experienced severe test anxiety. After the intervention, 29.8% (n = 17) still had severe test anxiety, and 52.6% (n = 30) had moderate anxiety. The McNemar’s test did not reveal a statistically significant difference in the intensity of test anxiety before and after the intervention in the control group. When comparing the intensity of test anxiety before the intervention between the study groups, the chi-square test did not show a statistically significant difference. However, after the study, a statistically significant difference was observed (P = 0.004) (Table 2).

**Table 1:** Demographic characteristics of study samples (N = 114)

Variables		Groups		*P-*value*
		Control	Intervention	
Age (y), Mean (SD)		21.9 ± 3.1	21.9 ± 3.2	0.999
Sex, n(%)	Male	29(50.9)	29(50.9)	0.234
	Female	28(49.1)	28(49.1)	
Marital status, n(%)	Single	54(94.7)	55(96.5)	0.734
	Married	3(5.3)	2(3.5)	
Residence, n(%)	Dormitory	30(52.6)	19(33.3)	0.531
	Private house	10(17.5)	16(28.1)	
	With family	17(29.8)	22(38.6)	

* Based on the independent sample t-test and chi-square test.

**Table 2:** Comparing the study groups in terms of mean test anxiety before and after intervention (N = 114)

Study groups	Before the intervention	After the intervention	Test result ^ǂ^
	Mean ± SD^§^	Mean ± SD	
Intervention	18.4 ± 5.3	13.3 ± 4.8	t = 5.85 *P* = 0.001
Control	16.4 ± 6.4	16.4 ± 6.1	t = − 0.05 *P* = 0.963
Test result^€^	t = 1.85 *P* = 0.067	t = − 3.0 *P* = 0.003	

Note: ^§^ Standard deviation; ^ǂ^ Comparison was done by paired t-test; ^€^Comparison was done by independent sample t-test.

**Table 3:** Comparison of test anxiety levels before and after the intervention in study groups (N = 114)

Groups	Test anxiety levels	Before intervention, n(%)	After intervention, n(%)	*P*-value*
Intervention	Low (0–12)	7 (12.3)	30 (52.6)	0.001
	Moderate (13–20)	32 (56.1)	21 (36.9)	
	Sever (21–37)	18 (31.6)	6 (10.5)	
Control	Low (0–12)	8 (14.0)	10 (17.6)	1.000
	Moderate (13–20)	34 (59.7)	30 (52.6)	
	Sever (21–37)	15 (26.3)	17 (29.8)	
*P*-value*		0.103	0.004	

* Based on the McNemar test and chi-square test.

## Discussion

This trial aimed to examine the impact of auricular acupressure on test anxiety among medical students. Consistent with prior research [21, 25, 36–38], a significant proportion of participants in the current study experienced moderate to severe test anxiety. Test anxiety, classified as a form of situational anxiety, is highly prevalent among medical students [21] and is associated with detrimental physical symptoms (e.g., heart palpitations and nausea), psychological manifestations (e.g., agitation and insecurity), and academic consequences (e.g., academic underachievement) [27]. Factors contributing to test anxiety include academic procrastination, low self-confidence, inadequate preparation, sleep disturbances on the exam night, and poor time management [12–15, 27]. The correlation between test anxiety and suboptimal academic performance has been established in previous studies [29, 39]. Thus, intervention strategies are crucial for effectively managing this condition.

The findings of the study indicated that Shen Men auricular acupressure had a significant effect in reducing test anxiety. This outcome is consistent with the findings of Lee et al. (2021), where the intervention group received bilateral acupressure at the Shen Men point and the endocrine point, while the control group received a placebo intervention at the Sham point. The results revealed a notable decrease in both test anxiety and state anxiety, although no significant change was observed in trait anxiety [19]. Another study conducted in 2015, focusing on the impact of acupressure on test anxiety in nursing students, also demonstrated the effectiveness of acupressure in reducing test anxiety [24]. Furthermore, in a study by McFadden et al. (2012) involving 109 students, the effects of active acupressure, placebo acupressure, and soothing sounds were compared, and all three interventions demonstrated similar effectiveness [26]. A clinical trial conducted in 2020 examined the effects of two interventions, auricular stimulation, and expressive writing, on test anxiety among medical students. In the auricular stimulation group, needles were inserted in specific areas innervated by the vagus nerve one day prior to the test and remained in place until after the test. Students were instructed to press the needles for 3 to 5 min if they experienced anxiety. On the other hand, the expressive writing group was asked to write a handwritten essay lasting 15–20 min the day before the exam. The results indicated that auricular stimulation significantly reduced test anxiety, while expressive writing had no effect [21]. In another clinical trial by Chueh et al. (2018), the impact of auricular acupressure on anxiety, depression, and sleep quality among nursing students was investigated. Using a magnetic pellet, the Shen Men acupoint was continuously pressed for a duration of 4 weeks. The study observed recovery rates of 26.7% for sleep quality, 43.5% for anxiety, and 25% for depression [39]. Furthermore, a single-blind trial involving 68 nursing students examined the effect of acupressure on clinical stress. In this trial, the Shen Men point on the wrist and the Yintang point between the eyebrows were pressed for 30 min in 5-minute intervals. The results demonstrated a significant reduction in clinical stress [20]. The existing literature supports the findings of our study and highlights the effectiveness of acupressure in reducing situational anxiety among students. The precise mechanism underlying acupressure is not fully understood. This non-invasive form of acupuncture involves applying gentle pressure to specific reflex points on various parts of the body, such as the ears, head and neck, and abdomen. Stimulation of these reflex points regulates the function of the sympathetic and parasympathetic nervous systems, thereby improving overall bodily function and promoting a sense of comfort [40, 41]. Additionally, acupressure activates C tactile fibers, which elicit the “limbic touch” response, triggering hormonal and emotional reactions [20].

### Limitations

When applying the results of this study, several limitations should be taken into consideration. Firstly, the mental state of participants during acupressure could have potentially influenced the study outcomes, which was beyond the control of the researcher. Secondly, the study was conducted solely on university students, which may impact the generalizability of the findings to a broader population. Additionally, in this study, therapists of the same gender were utilized for both male and female students, potentially introducing a bias in the results. To address this limitation, both therapists underwent comprehensive training with an acupuncturist. Lastly, it’s important to note that the research findings are solely based on a subjective anxiety scale, as objective assessments were not included in the study.

## Conclusion

This study demonstrated the effectiveness of auricular acupressure in reducing test anxiety among medical students. Therefore, the use of this non-invasive, safe, easy-to-administer, and effective method is recommended for managing test anxiety in the student population. However, it is important to consider the limitations of relying solely on subjective anxiety scales. Further research utilizing objective measures is necessary to validate the effectiveness of Shen Men acupressure in reducing test anxiety.

## Data Availability

The datasets used and/or analyzed during the current study are available from the corresponding author on reasonable request.

## References

[1] Huynh HP, Sramek KN, Sifuentes KA, Lilley MK, Bautista EM. Keep Calm and Be Humble: Can Intellectual Humility Predict Test Anxiety? Psychological Reports. 2022:00332941221103524. 10.1177/00332941221103524 35617130

[2] Balwan WK, Kour S (2022). Test anxiety research: twenty First Century in Retrospect. J Adv Educ Philos.

[3] Putwain DW, von der Embse NP (2021). Cognitive–behavioral intervention for test anxiety in adolescent students: do benefits extend to school-related wellbeing and clinical anxiety. Anxiety Stress & Coping.

[4] Németh L, Bernáth L. The nature of cognitive test anxiety: an investigation of the factor structure of the cognitive test anxiety scale. Educational Assess. 2022:1–21.

[5] Hafezi A, Etemadi S (2022). Understanding the causes, factors, and methods of reducing students’ exam anxiety in high school exams. J Social Humanity Educ.

[6] Tsegay L, Shumet S, Damene W, Gebreegziabhier G, Ayano G (2019). Prevalence and determinants of test anxiety among medical students in Addis Ababa Ethiopia. BMC Med Educ.

[7] Bischofsberger L, Burger PH, Hammer A, Paulsen F, Scholz M, Hammer CM (2021). Prevalence and characteristics of test anxiety in first year anatomy students. Annals of Anatomy-Anatomischer Anzeiger.

[8] Da A, Agyei Fb (2021). Analysis of licensure examination anxiety and its influencing factors among undergraduate nursing students. J Integr Nurs.

[9] Qalawa SAA, Soliman MT (2021). Association between nurses’ Student’s quality of life and anxiety of exams in selected Universityat KSA. Editorial Board.

[10] Mastour H, Ghalibaf AM, Niroumand S. Remote online test anxiety during the Coronavirus Disease 2019 Crisis: a cross-sectional study among medical students. Iran Red Crescent Med J. 2022;24(3).

[11] Bolbolian M, Asgari S, Sefidi F, Zadeh AS. The relationship between test anxiety and academic procrastination among the dental students. J Educ Health Promotion. 2021. 10. 10.4103/jehp.jehp_867_20 PMC8057172 34084814

[12] Alammari MR, Bukhary DM (2019). Factors contributing to prosthodontic exam anxiety in undergraduate dental students. Adv Med Educ Pract.

[13] Al-Sahman LA, Al-Sahman RA, Joseph B, Javali MA. Major factors causing examination anxiety in undergraduate dental students-a questionnaire based cross-sectional study. Annals of Medical and Health Sciences Research. 2019;9(6).

[14] Patil SG, Aithala MR. Exam anxiety: its prevalence and causative factors among Indian medical students. Natl J Physiol Pharm Pharmacol 2017;7(12).

[15] Baytemir K. Do parents have exam anxiety, too? The predictive role of irrational beliefs and perfectionism with parental exam anxiety in explaining students’ exam anxiety. School Psychol Int. 2022:01430343221122387.

[16] Stanley TB, Ferretti ML, Bonn-Miller MO, Irons JG, Double-Blind A. Randomized, Placebo-controlled test of the effects of Cannabidiol on experiences of test anxiety among College Students. Cannabis and Cannabinoid Research; 2022. 10.1089/can.2022.0062 35861792

[17] Balon R, Starcevic V. Role of benzodiazepines in anxiety disorders. Anxiety Disorders. 2020:367–88. 10.1007/978-981-32-9705-0_20 32002938

[18] Guina J, Merrill B (2018). Benzodiazepines I: upping the care on downers: the evidence of risks, benefits and alternatives. J Clin Med.

[19] Lee E, Park J-H. Effect of acupressure on pre-exam anxiety in nursing students. Altern Ther Health Med. 2021:AT6370. 34331752

[20] Yildirim D, Akman Ö (2021). The effect of acupressure on clinical stress management in nursing students: a randomised controlled study. J Acupunct Meridian Stud.

[21] Usichenko T, Wenzel A, Klausenitz C, Petersmann A, Hesse T, Neumann N (2020). Auricular stimulation vs. expressive writing for exam anxiety in medical students–A randomized crossover investigation. PLoS ONE.

[22] Beikmoradi A, Najafi F, Roshanaei G, Esmaeil ZP, Khatibian M, Ahmadi A. Acupressure and anxiety in cancer patients. Iran Red Crescent Med J. 2015;17(3). 10.5812/ircmj.25919 PMC4441788 26019908

[23] Rizi MS, Shamsalinia A, Ghaffari F, Keyhanian S, Nabi BN (2017). The effect of acupressure on pain, anxiety, and the physiological indexes of patients with cancer undergoing bone marrow biopsy. Complement Ther Clin Pract.

[24] Olshan-Perlmutter M, Carter K, Marx J (2019). Auricular acupressure reduces anxiety and burnout in behavioral healthcare. Appl Nurs Res.

[25] Joseph V (2015). Effect of self acupressure on anxiety among the nursing students undertaking their university examination in a selected nursing college, Tamil Nadu, India. Indian J Appl Res.

[26] Klausenitz C, Hacker H, Hesse T, Kohlmann T, Endlich K, Hahnenkamp K (2016). Auricular acupuncture for exam anxiety in medical students—a randomized crossover investigation. PLoS ONE.

[27] McFadden KL, Healy KM, Hoversten KP, Ito TA, Hernández TD (2012). Efficacy of acupressure for non-pharmacological stress reduction in college students. Complement Ther Med.

[28] Krispenz A, Gort C, Schültke L, Dickhäuser O (2019). How to reduce test anxiety and academic procrastination through inquiry of cognitive appraisals: a pilot study investigating the role of academic self-efficacy. Front Psychol.

[29] Mahboudi HR (2019). A comparative study of test anxiety between students of Biological Sciences and Humanities at University College of Rub-Bi Rashid. J Appl Linguistics Appl Literature: Dynamics Adv.

[30] Cuschieri S (2019). The CONSORT statement. Saudi J Anaesth.

[31] Cho Y, Joo J-M, Kim S, Sok S (2021). Effects of Meridian Acupressure on stress, fatigue, anxiety, and self-efficacy of Shiftwork nurses in South Korea. Int J Environ Res Public Health.

[32] Sarason IG (1975). Test anxiety and the self-disclosing coping model. J Consult Clin Psychol.

[33] Raju PM, Mesfin M, Alia E (2010). Test anxiety scale: reliability among Ethiopian students. Psychol Rep.

[34] Rasouli Khorshidi F, Sarami G, Naderi H, Shojaei AA (2019). Modeling motivation in the relationship between test anxiety and academic procrastination with achievement. Educ Strategies Med Sci.

[35] Huang L, Huang W (2007). Handbook of auricular treatment prescriptions & formulae.

[36] Cipra C, Müller-Hilke B (2019). Testing anxiety in undergraduate medical students and its correlation with different learning approaches. PLoS ONE.

[37] Afzal H, Afzal S, Siddique SA, Naqvi S (2012). Measures used by medical students to reduce test anxiety. JPMA The Journal of the Pakistan Medical Association.

[38] Vatankhah M, Kalani N, Rayat Dost E, Abiri S (2018). Investigation of anxiety test in Medical students of Jahrom University of Medical Sciences: a cross-sectional, descriptive study. J Biochem Tech.

[39] Rana R, Mahmood N (2010). The relationship between test anxiety and academic achievement. Bull Educ Res.

[40] Chueh K-H, Chang C-C, Yeh M-L (2018). Effects of auricular acupressure on sleep quality, anxiety, and depressed mood in RN-BSN students with sleep disturbance. J Nurs Res.

[41] Au DW, Tsang HW, Ling PP, Leung CH, Ip P, Cheung W (2015). Effects of acupressure on anxiety: a systematic review and meta-analysis. Acupunct Med.

